# A Case of Tetanus Cured with Antitoxin

**Published:** 1896-01

**Authors:** George Jobson

**Affiliations:** Oil City, Pa.


### REPORTS OF CASES.

### A CASE OF TETANUS CURED WITH ANTITOXIN.

By GEORGE JOBSON, V.S.,
OIL CITY, PA.

The curative treatment by means of antitoxin, although confined as yet to a limited number of diseases, opens a field whose limits we cannot yet define; and many diseases which are now looked upon as fatal may, in the near future, be cured by this form of treatment.

The discovery of tetanus antitoxin is one of the most recent and valuable additions to the list, and it is the only known remedy at present that can be relied upon to cure this generally fatal disease. Before proceeding with a description of the case referred to above, I will give a brief outline of the etiology of the disease, so that we may better understand the action of the remedy.

Tetanus is a germ disease which attacks most all species of domestic animals and man, and is characterized by spasmodic convulsions of the muscles, which before attacking the entire apparatus generally begin in a limited number; as, for instance, when the face muscles are affected and the jaws become locked it is called lockjaw. Later, the entire apparatus is invaded and the body becomes rigid—a condition which is brought about by the entrance of germs into the system, and the formation by them of poisons called toxins. That tetanus occurs spontaneously has very few supporters at present, as it is necessary to have the presence of the toxin before the disease is produced, and the germ which produces it may gain entrance to the system by the slightest kind of a wound. The bacillus of tetanus is an anaërobic germ, *i. e.*, one which cannot live in the presence of the atmosphere, as the oxygen which it contains is destructive to it. The germ is widely diffused to all parts of the earth, and occurs in manure, rich garden earth, and, in fact, anywhere the air does not penetrate. It is said its presence has been revealed in the intestines of herbivora, and this may be an explanation of the cause of the so-called cases of idiopathic tetanus, the enemy gaining entrance by means of a wound in the intestinal wall.

When the tissues are bruised and torn their vitality is lessened and a condition exists which is favorable to the growth of the bacillus; but should the wound be clean-cut and deep, and the arterial blood flow freely, the conditions are not so favorable, as the oxygen contained in the blood tends to destroy the germs, and the healthy tissue surrounding the wound sends forth its hosts of migratory cells to combat the enemy. When the bacilli find a medium suitable for their growth they secrete a poison or toxin, which is the element that causes the symptomatic muscular contractions. The bacillus does not then cause the tetanic convulsions, except in an indirect manner; that is, by the production of the poison or toxin, which acts as an irritant. If the system possesses sufficient vitality to resist the action of the toxin for a length of time, a material called antitoxin is formed by it, which neutralizes the action of the former. If, then, we supply the system with antitoxin, we fortify it with the material it craves to combat the germ poison, so that by the use of prepared antitoxin we can supply it in several days with a material which it requires months to form, granted it can resist the action of the virulent poison of the germs so long.

The case about to be described is not cited simply because the animal recovered, as I have had several cases recover by medicinal treatment alone, but because the effects of the material were almost immediate, while by the old methods it required months sometimes to produce the same results:

CASE. A chestnut pony gelding, used in a grocery wagon, received a wound in the neck from a rusty nail sticking out of a board in the stall. In about nine days afterward the man in charge noticed a slight stiffness during locomotion, but it was not thought serious, and the animal was kept at work until the next day (October 29th), when he was brought to the office and a diagnosis of incipient tetanus was made, and it was advised to have the animal unhitched and removed to his stall. Nerve-sedatives and antispasmodics were given, but the animal grew steadily worse, and on November 3d it was a well-marked case of tetanus. The muscles of the trunk and limbs were now stiff, but trismus had not as yet occurred, and with assistance the animal was able to rise when down.

Having read an article by Paul Gibier, M.D., of the Pasteur Institute, on the treatment of this disease in man by the use of antitoxin, I suggested to the owner that we send for a sufficient quantity to give it a fair trial, if it did not cost too much, and he consented. Before the arrival of the antitoxin, on the morning of November 7th, the horse was found lying in the stall, unable to rise. Slings were put under him, and, on being raised to his feet, he was able to stand. The entire muscular system was stiff and the jaws locked. Respiration was rapid and conducted with much difficulty. The slightest disturbance would bring on spasms, and it looked doubtful if the horse would live any length of time. On November 9th a supply of antitoxin sufficient for three injections was received from the Pasteur Institute, and at 4.15 P.M., assisted by my father and S. Foster, M.D., the first injection was made. The hair being first shaved from portions of the neck, the parts were washed with a solution of carbolic acid, and the antitoxin was injected hypodermically. The pulse at that time was 44, temperature 100.6°, and respirations 72. The wound was washed with iodine 1, potassium iodide 2, and water 100 parts. When the second injection was given, next morning, the respiration had dropped to 55 per minute, pulse 40, and the temperature had risen to 101.6°. Improvement in the condition of the muscles of the body and limbs was general, while the muscles of the neck and jaws were decidedly improved, the neck being moved from side to side, instead of being held in a straight line as previously. On November 11th the third injection was made, and a further improvement was visible, the respiration being 52, pulse 40, and temperature 101.4°. The muscles of the jaws could be freely used in masticating. Improvement continued steadily until six days after, when he was taken out of the slings and walked around the yard. Recovery is now complete, and, with the exception of being slightly emaciated, the animal looks none the worse for the attack.

